# Preclinical evaluations of acellular biological conduits for peripheral nerve regeneration

**DOI:** 10.1177/2041731413481036

**Published:** 2013-02-28

**Authors:** I-Chien Liao, Hua Wan, Shijie Qi, Cunqi Cui, Paarun Patel, Wendell Sun, Hui Xu

**Affiliations:** 1 Department of Research & Development, LifeCell Corporation, Bridgewater, NJ, USA; 2 Notre Dame Hospital, CHUM, University of Montreal, Montreal, QC, Canada; 3 Allergan Medical, Goleta, CA, USA

**Keywords:** Peripheral nerve regeneration, decellularized tissue, nerve guide, mesenchymal stem cells, regenerative medicine

## Abstract

Various types of natural biological conduits have been investigated as alternatives to the current surgical standard approach for peripheral nerve injuries. Autologous nerve graft, the current gold standard for peripheral nerve damage, is limited by clinical challenges such as donor-site morbidity and limited availability. The purpose of this study was to evaluate the efficacy of using acellular xenographic conduits (nerve, artery, and dermis) for the repair of a 1.2 cm critical size defect of peripheral nerve in a rodent model. Four months post surgery, the animal group receiving acellular artery as a nerve conduit showed excellent physiological outcome in terms of the prevention of muscle atrophy and foot ulcer. Histological assessment of the bridged site revealed excellent axon regeneration, as opposed to the nonrepaired control group or the group receiving dermal conduit. Finally, the study evaluated the potential improvement via the addition of undifferentiated mesenchymal stem cells into the artery conduit during the bridging procedure. The mesenchymal stem cell–dosed artery conduit group resulted in significantly higher concentration of regenerated axons over artery conduit alone, and exhibited accelerated muscle atrophy rescue. Our results demonstrated that xenographic artery conduits promoted excellent axonal regeneration with highly promising clinical relevance.

## Introduction

Despite recent technological advancements, peripheral nerve injury remains a critical clinical issue, leading to functional loss in 2.8% of all trauma patients.^1,2^ There are 50,000 procedures performed annually to repair damaged peripheral nerves, with an associated annual cost of US$7b in the United States alone.^2^ Patients with peripheral neuropathy typically suffer from muscle weakness, loss of tactile senses, and increased likelihood to develop chronic ulcers. Shortly after axotomy, Wallerian degeneration occurs in the distal segment of the axons and leads to atrophy in the connected muscle. Schwann cells and macrophages function to remove cell debris and form bands of Büngner. The regenerating axon fibers track along the bands to innervate the original motor output, and are followed by the remyelination of the new axon fibers. Axon regeneration slowly progresses at a rate of 1.5 mm/day in humans, and any defect size greater than 3 cm often results in poor functional recovery.^3,4^

The current gold standard procedure to bridge damaged peripheral nerves is the use of autologous nerve grafts. However, drawbacks such as inadequate graft length, donor-site morbidity, and limited tissue availability led to the search for alternative solutions.^4–8^ The development of nerve conduits has matured significantly since the use of silicone tubes, which prevented axon regeneration.^9^ Current Food and Drug Administration (FDA)-approved nerve conduits largely consist of type I collagen (NeuraGen^®^ (Integra, Plainsboro, NJ) and NeuroMatrix^®^/Neuroflex^®^ (Stryker, Kalamazoo, MI)) or synthetic polymers (Neurotube^®^, NeuroLac^®^ (AxoGen, Inc, Alachua, FL), and SaluBridge (SaluMedica, Smyrna, GA)). There are encouraging reports regarding the use of collagen conduits to bridge short nerve defects (<2 cm), but recent studies reported insufficient axon regeneration when using collagen conduits to repair a 2.8-cm defect.^10^ Some of the drawbacks with synthetic nerve conduits include chronic inflammation, acidic degradation by-products, and rigidity mismatch, resulting in suboptimal functional recovery.^7,8,11,12^ Recent approaches to functionalize synthetic nerve conduits with neurotrophic factors and topographical cues require further clinical studies to demonstrate conclusive functionality.^4,6,13–17^ As an alternative strategy, biological tissue−derived conduits and bone marrow stem cells have been investigated for peripheral nerve regeneration.^10,18–25^ Bone marrow stem cells have been shown to be capable of differentiating into neuronal and glial phenotypes, and the use of bone marrow stem cells in a collagen gel−filled vein was shown to bridge a 1.5-cm defect effectively.^23^

The focus of this study was to investigate the regenerative efficacy of various types of acellular biological conduits for severed peripheral nerve in a rat model. This study evaluated three different types of tissues in their ability to bridge a 1-cm critical size nerve gap: nerve (acellular processed nerve (APN)), artery (vascular conduit (VC)), and dermal tissue (acellular dermal tissue (ADM)). The regenerative outcome was compared against an unrepaired control group (defect only (DEF)) and an autograft control group (autograft (AUTO) at 4 months post surgery. Finally, undifferentiated mesenchymal stem cells (MSCs) were injected into the artery conduits (VC with MSCs (VC-MSCs)) to determine the potential of improvement due to stem cell incorporation.

## Materials and methods

### Tissue isolation and processing to severed nerve conduits

Three different types of decellularized conduits were compared in their ability to facilitate sciatic nerve repair: nerve (APN), artery (VC), and dermal tissue (ADM). The small branches of carotid artery (~1 mm in diameter) and small neck vagus nerves were harvested from pigs (Large White Landrace, Farm to Pharm LLC, Warren, NJ). The porcine skin was harvested and procured to a layer of 0.5 mm with an intact basement membrane. The tissues were processed using LifeCell Corporation’s proprietary processing method (Branchburg, NJ).^26–29^ Briefly, the acquired tissues were incubated with 1% Triton X-100 in RPMI with 25 mM ethylenediaminetetraacetic acid and 1% gentamicin to solubilize and remove cells and cell remnants, and enzyme-treated Pulmozyme^®^ 30 U/mL for DNA removal (Genentech Inc., South San Francisco, CA), and maintained in aseptic conditions prior to implantation. The dermal tissue was sterilized with electron beam irradiation (dosage of 15 kGy) and was sutured into a tubular structure to serve as a nerve conduit prior to implantation. The human dermal tissue used in this study was obtained from LifeCell Corporation, which is regulated by the American Association of Tissue Banks. The obtained human dermal tissue was processed in a similar manner as the porcine dermal tissue as described above. All of the conduits used in this study ranged from 1–2 mm in diameter.

### Conduit histology and immunohistochemistry evaluation

The nerve conduits were stained with hematoxylin and eosin (H&E), Verhoeff’s stain, and alcian blue to evaluate the tissue structure and elastin/glucosaminoglycan content. The nerve conduits were further immunostained for collagen I (MD Biosciences, St. Paul, MN), collagen III (Millipore, Billerica, MA), collagen IV (Millipore), laminin (Abcam, Cambridge, MA), and fibronectin (Abcam). During evaluation of explants, the middle portion of explanted grafts was fixed in 10% formalin and processed for H&E and osmium tetroxide staining. The presence of neural axons through the nerve guide was verified using mouse antineurofilament 200 antibody (Sigma–Aldrich, St. Louis, MO).

### Bone marrow stem cell isolation and culture

Bone marrow stem cells were isolated from the femurs and tibia of adult male Lewis rats as previously described.^30^ The presence of MSCs was validated through positive immunostaining of integrin β1, CD54, and negative staining for CD14 and CD45 (data not shown). The multipotency of the MSCs was demonstrated using MSC osteogenesis and adipogenesis kits (Millipore). The osteogenic and adipogenic differentiations were confirmed with Alizarin Red S and Oil Red O staining, respectively (data not shown). The MSCs were expanded in culture, maintained in an undifferentiated state and incorporated into the VC-MSC conduits at a concentration of 5 million cells/conduit.

### Animal study design/surgical procedure

Adult male Lewis rats (9–11 weeks old; Charles River Laboratories, Wilmington, MA) were used in this study. The animals were organized into six experimental groups (as defined earlier): DEF (n = 3); AUTO (n = 3), APN (n = 5), VC (n = 5), VC-MSCs (n = 5), and ADM (n = 9), including human (n = 6) and porcine (n = 3) acellular dermal conduits. To evaluate if the basement membrane was necessary for the nerve regeneration, human acellular dermal conduits were created to render the basement membrane facing inward (n = 3) or outward of the bridged nerve stumps (n = 3). In this study, defect and autograft groups served as negative and positive controls, respectively.

The animals were anesthetized, and incisions were made at the gluteal and posterior thigh to expose the sciatic nerve to the left lower biceps femoris. A 12-mm defect of the sciatic nerve was surgically created. The DEF group had the excised nerve left unrepaired, while the excised nerve for the AUTO group was immediately sutured back after the defect creation. For the APN, ADM, and VC groups, the proximal and distal axons were bridged with the respective conduits. For the VC-MSCs group, 5 million cells were concentrated into a 25 µL cell suspension and injected into each artery conduit before suturing the conduit with the nerve stump. The animals were maintained under veterinarian supervision with standard rodent diet and water, along with 12-h day/night cycle for 4 months. All animal care in this study was in compliance with the institutional guidelines of North American Science Associates, Inc. (Northwood, OH).

### Study outcome and statistical analysis

The recovery of the lower left leg functions as a result of sciatic nerve damage was assessed via a broad category of tests: withdrawal reflex to pain stimulation performed by a needle puncture, strephexopodia, walking locomotion (presentation of limping motion during walking based on clinical observation), measurement of circumference of the lower left and right legs, and presentation of foot ulcer and autotomy. Autotomy (self-amputation of digits that have lost sensory response) provides an indication of the extent of physiological functional recovery to the affected joints. To document the difference in the extent of autotomy, the finger prints were collected after the animals were euthanized via CO_2_ gas. The behavioral analysis was conducted weekly until 4 months post surgery. To evaluate the amount of muscle mass recovery due to the repair of sciatic nerve, the gastrocnemius muscles of the animals were excised to obtain their dry weight. The weight of the muscle from the repaired lower left leg was normalized to the undamaged contralateral leg.

All data (mean ± standard error of mean (SEM)) were analyzed using Kruskal–Wallis analysis with Bonferroni correction followed by Tukey’s test for statistical significance. The analysis was performed on Minitab 16 statistical software. *P* values less than 0.05 were considered to be statistically significant.

## Results

### Structural properties of acellular nerve conduits

**Figure 1.**
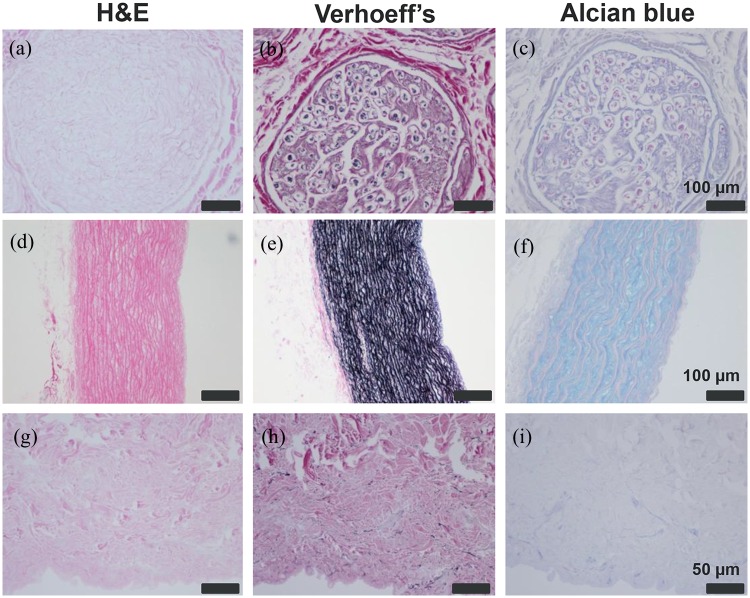
Histology of acellular biological conduits. H&E, Verhoeff’s and Alcian blue staining of (a, b, and c) APN, (d, e, and f) ADM, and (g, h, and i) VC conduits. No cellular components were detectable after decellularization; the extracellular matrix structures were well preserved. Scale bars are 100 µm for (a), (b), (c), (d), (e), and (f) and 50 µm for (g), (h), and (i). ADM: acellular dermal matrix conduit; APN: acellular processed nerve conduit; H&E: hematoxylin and eosin stain; VC: vascular artery conduit.

Decellularized nerve (APN), artery (VC), and dermis (ADM) conduits were evaluated histologically for their tissue extracellular matrix (ECM) content. After decellularization, the lumen structure of the APN conduit was largely intact. Histological assessment of the tissue revealed intact epineurium and perineurium structures, while the endoneurium was stripped away after processing (Figure 1(a) to (c)). Immunostaining suggested that the APN conduits were rich in collagen types I and IV and laminin, yet negative for collagen type III and fibronectin (see Appendix 1, Figure 6(a) to (e)). Decellularized artery (VC) conduits showed a hollow lumen with intact layers of tunica intima, media, and adventitia (Figure 1(d) to (f)). The VC conduits retained their elastic fibers, which prevented the artery vessel from collapsing after implantation (Figure 1(e)). Similar to the APN conduits, the VC conduits were positive for types I and IV collagen and laminin, but negative for collagen type III and fibronectin (see Appendix 1, Figure 6(f) to (j)). The dermis (ADM) conduits also had hollow lumen and largely consisted of collagen types I and III, laminin, and fibronectin (see Appendix 1, Figure 6(g) to (i) and (k) to (o)). All decellularized conduits were devoid of any cellular nucleus and had no measurable DNA remnant (data not shown).

### Muscle atrophy and behavioral analysis

**Figure 2.**
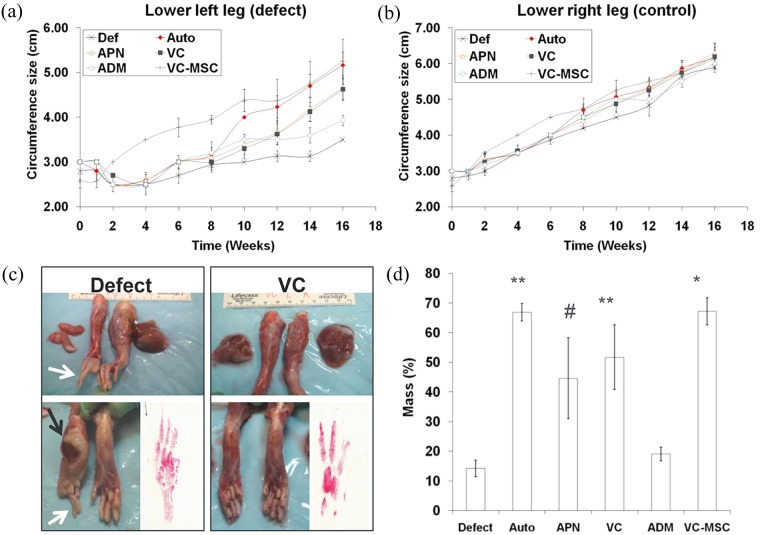
Alleviation of muscle atrophy and foot ulcers. Measurement of the circumference size of (a) the lower left leg affected by the sciatic nerve resection and (b) the unaffected lower right leg over 4 months. (c) Gross images of the affected left legs showed rescue of muscle atrophy and foot ulcer in the VC group compared with the DEF group. (d) The wet mass of the gastrocnemius muscle of the affected left leg was compared to the unaffected right leg. The difference in the wet mass of the gastrocnemius muscle quantifies the extent of muscle atrophy rescue. ADM: acellular dermal matrix conduit; APN: acellular processed nerve conduit; DEF: unrepaired defect; VC: vascular artery conduit; VC-MSCs: vascular artery conduit with mesenchymal stem cells. *p < 0.05 versus DEF, ADM, and APN; **p < 0.05 versus DEF and ADM; and ^#^p < 0.05 versus DEF, ADM, and VC-MSCs.

The physiological recovery from peripheral nerve damage was determined by a weekly assessment of an array of physiological tests (painful reflex, strephexopodia, and walking pattern). Table 1 shows the number of weeks required for the regaining of physiological function (painful reflection, strephexopodia, and walking), as well as the development of autotomy and food ulcer. AUTO and VC-MSCs groups had recorded physiologic recovery of painful reflection and walking at 14 weeks post surgery, while the APN and VC groups recovered at 16 weeks post surgery (Table 1). DEF and ADM groups, however, never recovered in the 4-month study period (Table 1). The circumference size of the affected lower left leg and unaffected lower right leg was recorded over time as an indication of recovery from muscle atrophy (Figure 2(a) and (b)). At 16 weeks post surgery, DEF and ADM groups recorded much smaller circumference sizes and gastrocnemius muscle mass compared with the AUTO group (Figure 2(a) and (d)). The circumference size and muscle mass of the VC and APN groups were not statistically significantly different compared with the AUTO group. Interestingly, the VC-MSCs group had the highest circumference size, and the muscle mass was equivalent to the AUTO group (Figure 2(a) and (d)). The development of autotomy (white arrow, Figure 2(c)) and foot ulcer (black arrow) were obvious in the DEF group but not seen in the VC group. Foot ulcers and autotomy were observed in both the DEF (2 of 3 animals) and ADM (7 of 9 animals) groups. In contrast, the AUTO, APN, VC, and VC-MSCs groups did not develop ulcer and autotomy (Table 1, Figure 2(c)). No difference in terms of the functional outcomes was found between human and porcine dermal conduits. Similarly, the inward/outward orientation of the basement membrane of dermal tissue conduits did not show appreciable benefit in terms of the functional recovery.

**Table 1.** Behavioral and physiological analysis.

Group	Painful reflection	Autotomy	Foot ulcer	Strephexopodia	Walking (limp)
DEF	No	2/3	2/3	No recovery	No recovery
AUTO	12w	0/3	0/3	Recovered at 14w	Recovered at 14w
ADM	No	1/3	1/3	No recovery	No recovery
APN	16w	0/5	0/5	Recovered at 16w	Recovered at 16w
VC	16w	0/5	0/5	Recovered at 16w	Recovered at 16w
VC-MSCs	14w	0/5	0/5	Recovered at 14w	Recovered at 14w

ADM: acellular dermal matrix conduit; APN: acellular processed nerve conduit; AUTO: autograft; DEF: unrepaired defect; VC: vascular artery conduit; VC-MSCs: vascular artery conduit with mesenchymal stem cells; w: number of weeks post surgery.

### Histological assessments of peripheral nerve regeneration

**Figure 3.**
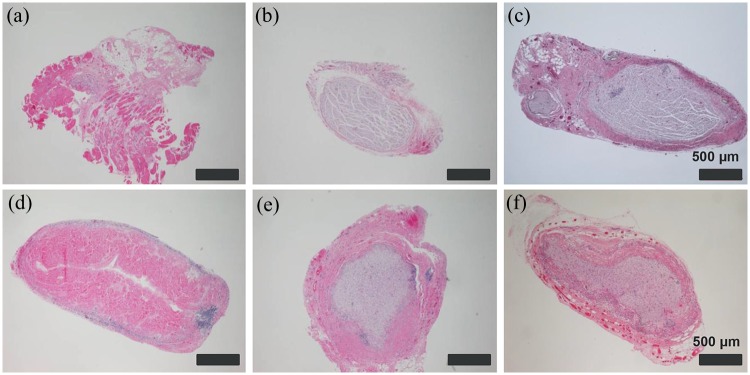
Histology of implanted conduits at 4 months. H&E staining of the (a) DEF, (b) AUTO, (c) APN, (d) ADM, (e) VC, and (f) VC-MSCs) groups at 4 months post surgery. Significant level of neuronal structures filled the lumen of the conduit in the AUTO, APN, VC, and VC-MSCs groups. The ADM group was filled with connective tissue, while the resected nerve of the DEF group remained unrepaired. Scale bar: 500 µm. ADM: acellular dermal matrix conduit; APN: acellular processed nerve conduit; AUTO: autograft; DEF: unrepaired defect; H&E: hematoxylin and eosin stain; VC: vascular artery conduit; VC-MSCs: vascular artery conduit with mesenchymal stem cells.

**Figure 4.**
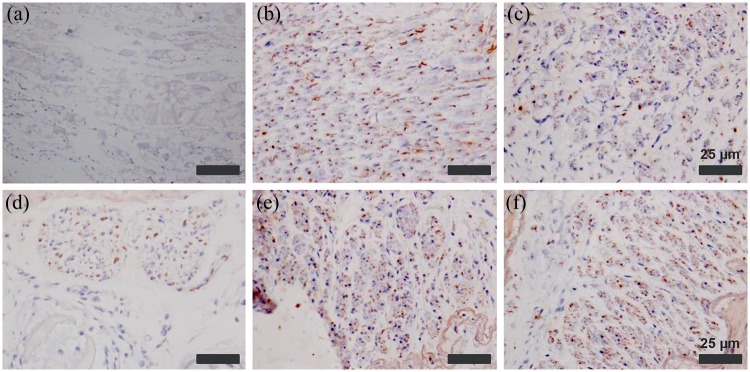
Immunostaining for neurofilaments in the implanted conduits. Anti-NF 200 staining of the (a) DEF, (b) AUTO, (c) APN, (d) ADM, (e) VC, and (f) VC-MSCs groups at 4 months post surgery. There were significant amounts of positively stained neurofilaments in the lumen of the conduit in the AUTO, APN, VC, and VC-MSCs groups as opposed to the DEF and ADM groups. Scale bar: 25 µm. ADM: acellular dermal matrix conduit; anti-NF: antineurofilament; APN: acellular processed nerve conduit; AUTO: autograft; DEF: unrepaired defect; VC: vascular artery conduit; VC-MSC: vascular artery conduit with mesenchymal stem cells.

**Figure 5.**
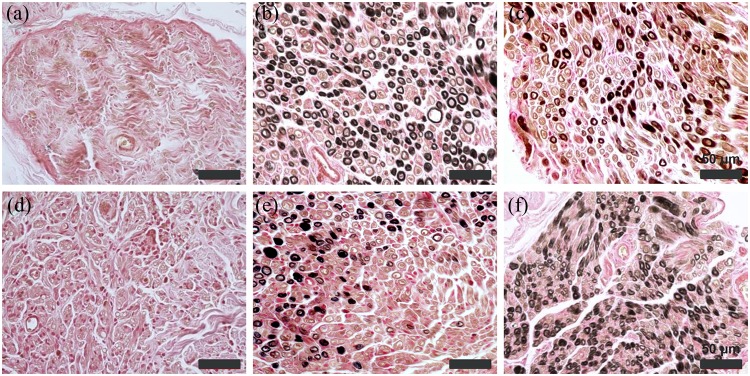
Osmium staining for myelinated axons in the implanted conduits. Osmium staining of the (a) DEF, (b) AUTO, (c) APN, (d) ADM, (e) VC, and (f) VC-MSCs groups at 4 months post surgery. There were significant levels of myelinated axons in the lumen of the conduit in the AUTO, APN, VC, and VC-MSCs groups as opposed to the DEF and ADM groups. Scale bar: 50 µm. ADM: acellular dermal matrix conduit; APN: acellular processed nerve conduit; AUTO: autograft; DEF: unrepaired defect; VC: vascular artery conduit; VC-MSCs: vascular artery conduit with mesenchymal stem cells.

**Figure 6.**
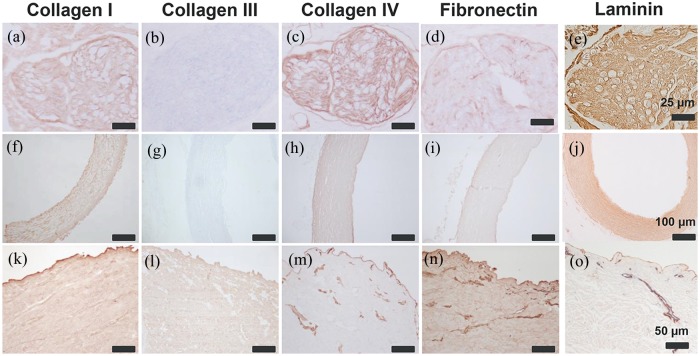
Immunostaining of acellular biological conduits. Collagen I, collagen III, collagen IV, fibronectin, and laminin staining of the (a, b, c, d, and e) APN, (f, g, h, i, and j) ADM, and (k, l, m, n, and o) VC conduits. APN and VC conduits both positively stained for laminin and collagen types I and IV and negatively stained for collagen III and fibronectin. The ADM conduit, however, stained more strongly for collagen III and fibronectin but negatively stained for laminin. Scale bar: 50 µm. ADM: acellular dermal matrix conduit; APN: acellular processed nerve conduit; DEF: unrepaired defect; VC: vascular artery conduit.

**Figure 7.**
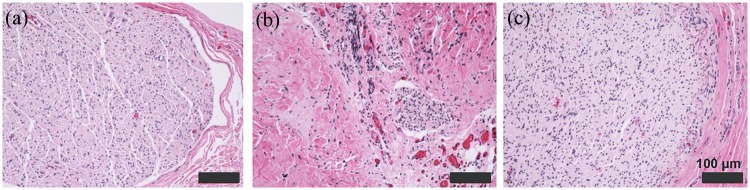
Histological assessment of the biological conduits. H&E staining of the (a) AUTO, (b) ADM, and (c) VC groups at a higher magnification. Significant level of axon growth was apparent in the AUTO and VC groups but not in the ADM group. Scale bar: 100 µm. ADM: acellular dermal matrix conduit; AUTO: autograft; DEF: unrepaired defect; H&E: hematoxylin and eosin stain; VC: vascular artery conduit.

**Figure 8.**
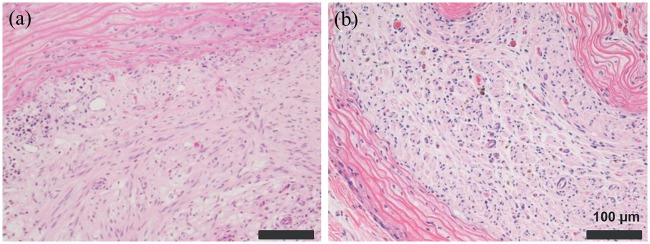
Histological assessment of the VC group at earlier time points. H&E staining of the VC group at (a) 1 month and (b) 4 months post surgery. Significant level of axon growth was absent at the 1-month time point but abundant at the 4-month time point. Scale bar: 50 µm. H&E: hematoxylin and eosin stain; VC: vascular artery conduit.

**Figure 9.**
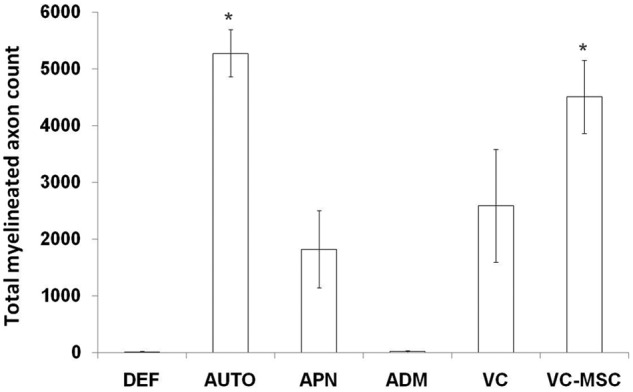
Myelinated axons in the repaired nerve at 4 months post surgery. The total number of myelinated axons in the bridged peripheral nerve were quantified for the repaired nerve at 4 months post surgery. The VC-MSCs and the AUTO groups were statistically significantly higher in the number of myelinated axons when compared against the DEF and ADM group. ADM: acellular dermal matrix conduit; APN: acellular processed nerve conduit; AUTO: autograft; DEF: unrepaired defect; VC: vascular artery conduit; VC-MSCs: vascular artery conduit with mesenchymal stem cells. *p < 0.05 versus DEF and ADM.

The extent of axon regeneration of the study groups was assessed using H&E, osmium, and neurofilament immunostaining. Bridging of the severed nerve never occurred in the DEF group, as reflected by the presence of connective tissue between the two nerve stumps (Figure 3(a)). The AUTO, APN, VC, and VC-MSC groups all had significant levels of axon regeneration in the lumen of the conduits (Figure 3(b), (c), (e), and (f); also see Appendix 1, Figure 7(a) and (c)). The axon regeneration was not noticeable at the 1-month time point for the VC group, but was apparent at the 4-month time point (see Appendix 1, Figure 8(a) and (b)). As a comparison, the ADM group was filled with connective tissue inside the lumen (Figure 3(d); also see Appendix 1, Figure 7(b)). The regeneration of neurofilaments was confirmed via immunostaining in the AUTO, APN, VC, and VC-MSCs groups but not in the DEF and ADM groups (Figure 4). Osmium staining of the remyelinated axons was abundant in the AUTO, VC, and VC-MSCs groups (Figure 5(b), (e), and (f)). The APN group had visibly less remyelinated axons (Figure 5(c)). The DEF and ADM groups did not stain positively for remyelinated axons (Figure 5(a) and (d)). When the number of myelinated axons for the repaired nerve site was quantified (Appendix 1, Figure 9), the VC-SMC group was similar to the AUTO group and statistically significantly higher than the DEF and ADM groups.

## Discussion

There are many important criteria to consider when designing a peripheral nerve conduit to achieve optimal regenerative outcome.^4,7,9^ First, the conduit must match the dimension and mechanical rigidity of a natural nerve, while possessing great biocompatibility and inducing limited foreign material response as the conduit degrades or resorbs. Naturally derived ECMs, such as collagen I and laminin, have a profound impact on the fate of the bridged nerve stumps and have been suggested to be stimulatory to neuronal cell growth.^9^ The use of synthetic materials as a nerve guide often leads to chronic foreign material response around the implant, whereas the presence of naturally existing ECM in the biological conduits provides important regenerative signaling cues in terms of cellular migration and repair. Finally, the material must allow adequate nutrient transport, provide neurotrophic factors, and present topographical cues to facilitate axon regeneration. In addition to properties that can be built into an acellular conduit, the addition of Schwann cells or stem cells is also promising in facilitating nerve repair. A recent study investigated the incorporation of Schwann cells, adipose stem cells, and MSCs into a fibrin conduit and demonstrated that the use of stem cells resulted in a significant increase in axon regeneration, although the mode of action remained unclear.^31^

In this study, biologically derived nerve conduits were evaluated for their potential in repairing damaged peripheral nerves. As observed in LifeCell Corporation’s previous proprietary technologies, the implanted xenographic conduits showed high preservation of their native ECM structure while eliciting very little immune rejection (Figure 3).^27^ In the VC conduit, the retention of elastic fibers eliminated the concern of graft collapse post surgery, while the preservation of collagen and laminin was important to encourage rapid nerve growth. All of the conduits compared in this study have similar mechanical rigidity as native nerve (data not shown), and no sign of degradation occurred in the test period of 4 months (see Appendix 1, Figure 7). The preserved nerve structure inside the lumen of the APN conduit appeared to have physically interfered with the rate of nerve fiber growth through the conduit, as shown in the reduced amount of new myelinated axons (Figure 5(c)). However, the VC and VC-MSC groups demonstrated promising results in the rescue of muscle atrophy and stimulation of myelinated axon regeneration (Figures 2, 4, and 5). The extent of nerve repair observed in these groups was at approximately the same level as that seen in the AUTO group. The VC-MSC group did appear to have a greater concentration of myelinated axons, but it is undetermined whether the MSCs differentiated into neural cells or secreted neurotrophic factors to enhance regeneration. The regenerative property of using artery as a nerve conduit was highlighted when compared to the ADM group, where the conduit lumen was filled with connective tissue instead (Figure 5; Appendix 1, Figure 9).

It has been demonstrated in this study that the use of decellularized arteries is a promising alternative strategy for peripheral nerve repair. The xenographic source of the biological conduits circumvented the limitation of graft availability and dimension, while the trans-species acceptance of the acellular graft was made possible via the removal of all cellular remnants and alpha galactose.^27^ The highly preserved ECM composition is rich in collagen I and laminin, thus serving as a ready-to-use, neurogenic conduit as opposed to an engineered collagen tube.^9^ Future studies to evaluate delineation and reinnervation of muscle by assessing muscle fiber type and cross-sectional area will provide in-depth understanding of muscle physiology during the time of recovery. As a definitive study, it will be very interesting to apply this technology into a higher level animal model with a larger nerve defect gap. Since most peripheral nerve damage occurs through trauma, this work demonstrated that the development of an off-the-shelf type of material would be ideal and highly translatable toward clinical applications.

## Conclusion

This study investigated the efficacy of using three different types of decellularized xenographic tissues (nerve, artery, and dermis) as peripheral nerve conduits to bridge a 1-cm defect gap of rat sciatic nerve. The processed tissues showed high preservation of their ECM structures and were stripped of all cell nuclei and inflammatory α-galactose. Animals receiving artery and artery + MSCs showed an excellent outcome regarding rescue from muscle atrophy and ulcer development, and facilitation of myelinated axon regeneration. The artery group induced nerve repair to a similar level as nerve autograft, and was significantly better than decellularized nerve or dermis conduits. This study demonstrated the promising potential of using artery as a ready-to-use biological conduit for patients challenged with peripheral nerve damage.
